# Safety and efficacy of interleukin-6-receptor inhibitors in the treatment of neuromyelitis optica spectrum disorders: a meta-analysis

**DOI:** 10.1186/s12883-021-02488-y

**Published:** 2021-11-23

**Authors:** Sanjeev Kharel, Suraj Shrestha, Rajeev Ojha, Neha Guragain, Rakesh Ghimire

**Affiliations:** 1 Maharajgunj Medical Campus, Tribhuvan University Institute of Medicine, P.O. Box: 44600, Kathmandu, Nepal; 2 Department of Neurology, Tribhuvan University Teaching Hospital, Maharajgunj, Kathmandu, 44600 Nepal; 3 Department of Clinical Pharmacology, Tribhuvan University Teaching Hospital, Maharajgunj, Kathmandu, 44600 Nepal

**Keywords:** Interleukin-6-receptor inhibitors, Drug therapy, NMO/NMOSD

## Abstract

**Background:**

Interleukin-6-receptor inhibitors like Tocilizumab and Satralizumab are showing promising results in the treatment of Neuromyelitis Optica spectrum disorder (NMOSD). We aimed to investigate the efficacy and safety of various Interleukin-6-receptor inhibitors in the management of NMO/NMOSD.

**Methods:**

PubMed, Embase, and The Cochrane Library were systematically searched for suitable studies. Change in Annualized Relapse Ratio (ARR), Change in Extended Disability Status Scale (EDSS) s, the proportion of relapse-free patients and proportion of patients with adverse events, including serious adverse events and mortality were the parameters considered for the meta-analysis for Tocilizumab. Mean difference (MD) with 95% CI was used to quantify the change in ARR and change in EDSS before and after treatment. A forest plot was prepared to indicate the efficacy and adverse effects outcomes. The results were compared with those of Satralizumab included in two trials.

**Results:**

A total of nine studies with 202 patients were included in our study. Tocilizumab found a good proportion (76.95% CI: 0.61–0.91; *p* < 0.001) of relapse free patients at follow up. It also significantly reduced mean ARR (mean difference: -2.6, 95% CI: − 2.71 to − 1.68; *p* < 0.001) and but did not show significant difference in change in EDSS score (mean difference = − 0.79, 95% CI: − 1.89 to − 0.31; *p* = 0.16). Also, the toxicity profile of Tocilizumab was acceptable considering the proportions of patients with adverse events 56% (95% C.I.;0.27–0.85, I^2^ = 88.95%, *p* < 0.001), proportions of patients with serious adverse events 11% (95% C.I.; 0.05 to 0.17, I^2^ = 0%, *p* < 0.001) and zero treatment related deaths. SAkura studies for Satralizumab showed similar relapse free patients (70% to 80%) and reduction of ARR and EDSS from baseline. Some studies of Tocilizumab have shown to reduce pain and fatigue while trials of Satralizumab had non-significant findings.

**Conclusion:**

Interleukin-6-receptor inhibitors therapy showed a promising result with good efficacy and acceptable adverse events profile for treatment of NMOSD.

**Supplementary Information:**

The online version contains supplementary material available at 10.1186/s12883-021-02488-y.

## Background

Neuromyelitis Optica Spectrum Disorder (NMOSD), previously called Devic’s disease is an Aquaporin-4-Immunoglobulin G (AQP4-IgG) antibody-associated autoimmune inflammatory disease of the Central Nervous System mostly involving the optic nerve and spinal cord [1]. Similarly, involvement of cerebrum, diencephalon, or brainstem are also frequently observed, in about 80% of patients [2]. Myelin oligodendrocyte glycoprotein (MOG) antibody found in AQP-4 negative NMOSD patients, has also been recently described [3]. Several studies have shown the prevalence rate of NMOSD ranging from 0.37 to 4.1 per 100,000 persons and up to 10 per 100,000 persons in certain racial groups [4, 5]. Females, people with age greater than 35 years, and Asian or African races are particularly at an increased risk for developing NMOSD [6].

The primary aim of treatment in NMOSD is to reduce the severity of acute attacks, prevent relapses, and maintain remission [7]. To achieve this, various groups of drugs have been used. For the prevention of relapses, immunosuppressive drugs such as azathioprine and mycophenolate mofetil are used and are also found effective. However, it comes with the cost of inevitable adverse effects because of prolonged or long-life immunosuppression [8, 9].

To counterfeit this issue, humanized recombinant monoclonal antibody drugs like eculizumab, inebilizumab, and satralizumab targeting different receptors like anti-CD-20, Interleukin-6(IL-6), complement-5(C-5), etc. are being widely used and studied [10]. Interleukin-6-receptor inhibitors like Tocilizumab and Satralizumab, are now being considered as good options for treatment of NMO/NMOSD and potential therapeutic effects of Tocilizumab and Satralizumab have been investigated via clinical trials and have shown promising results in the treatment of active NMOSD case, however, summarized data is lacking [11]. To establish Interleukin-6-receptor inhibitors drugs as efficacious and tolerated treatment options in the management of NMO/NMOSD, this meta-analysis is done with the aim of finding the combined effect size of their efficacy and safety from real-world studies.

## Methods

This systematic review and meta-analysis were carried out and reported according to the Preferred Reporting Items for Systematic reviews and Meta-Analyses (PRISMA) statement [12]. Our meta-analysis aims to explain the role of Interleukin-6-receptor inhibitors or Anti-Interleukin Receptor drugs (Tocilizumab and Satralizumab) for the treatment of patients with Neuromyelitis Optica Spectrum Disorder (NMOSD).

### Study registration and protocol

The study protocol, with well-defined methodology and inclusion criteria, was registered on PROSPERO with reference number ID: CRD42021226900.

### Inclusion and exclusion criteria

All original research studies in the English language published until December 5, 2020, discussing the efficacy and/or safety of Interleukin-6-receptor inhibitors (Tocilizumab, Satralizumab) administered in any doses (either low or high dose) and in any form (Intravenous or subcutaneous) for the treatment of NMOSD/NMO patients were considered eligible for inclusion. Studies reporting data on the use of these drugs given to patients of any age or nationality as monotherapy or in combination with other add-on therapies were included. The objective outcomes needed (at least one) in the study for inclusion were: Change in Annualized Relapse ratio (ARR), Change in EDSS score, the proportion of relapse-free patients, and proportion of patients with adverse events, including serious adverse events and mortality.

Studies involving any of these were excluded from the meta-analysis: 1) Studies with insufficient or unclear information 2) in vitro or animal studies 3) case reports, case series with ≤2 cases, conference abstracts, reviews, meta-analysis, editorials and commentaries, and 4) non-English studies.

### Search strategy and selection criteria

We searched PubMed, Embase and The Cochrane Library from the inception dates to December 5, 2020. Boolean logic was used for conducting a database search, and Boolean search operators “AND” and “OR” were used to link search terms. A combination of the following keywords was included: “neuromyelitis spectrum disorder”, “optic neuritis”, “NMOSD”, “Aquaporin 4 antibody”, “Devic’s disease”, “Anti-interleukin-6”, “anti-IL-6”, “IL6 receptor blockade”,” Tocilizumab” and “Satralizumab”. For advanced PubMed search, the medical subject headings (MeSH) database was used to find MeSH terms for the aforementioned search terms. Similarly, for advanced Embase search, Emtree terms were used for the aforementioned search terms. The search strategy is described in supplementary file 1. To find additional articles, manual searching of reference lists from selected articles was done. The search was also broadened to include preprint servers and thesis repositories while experts in the field were also inquired about ongoing studies. These additional searches were included in our analysis if they fulfilled our eligibility criteria.

### Data extraction

Two reviewers (SK and SS) imported all the above records to ENDNOTE v9 and ran duplicate searches. The duplicate records were then removed. Then, they evaluated remaining records by their titles and abstracts independently and assessed in detail the full texts of any potentially relevant articles against the eligibility criteria. Any disagreements or uncertainties were resolved through discussion with the help of a third author (RO). Two reviewers then independently extracted data from studies selected for inclusion, and any discrepancies resolved through discussion with help of a third reviewer (RO).

Following this, two reviewers (SK and SS) used a pre-designed standardized data extraction format to extract data under the following headings: Authors, year of publication, Interleukin-6-receptor inhibitors used, type of study, regions/countries where studies were conducted, sample size, follow-up period, number of females/males patients, mean age or range of patients, mean disease duration, number of Aquaporin-4 Ab positive patients, doses of drugs used, Add-on drugs and/or previously used drugs. The corresponding authors of the respective papers were contacted for clarification if required data were missing, not reported in the paper, or reported in an unusual form. Supplementary material associated with the main paper was also explored in cases whenever deemed necessary.

### Statistical analysis

The meta-analysis was conducted using the STATA software version 16 (StataCorp). A random-effects or fixed-effect model was used to pool the data, and statistical heterogeneity was evaluated using the I^2^ statistic. When I^2^ was ≤50%, a fixed-effect model was used for meta-analysis. When I^2^ was > 50%, DerSimonian, and Laird random-effects model was used for meta-analysis**.** Meta-analysis of the proportion of patients with relapse-free at last follow-up and proportion of patients with adverse events, serious adverse events were expressed as a pooled proportion with 95% confidence interval (CI). Meta-analysis for change in ARR and Change in EDSS-before and after treatment was expressed as a mean difference (MD) with 95% CI. While meta-analysis for on-trial relapse risk among randomized control trials (RCTs) studies was expressed as pooled Risk ratio between the intervention group and placebo group. Forest plots with 95% CIs were created to show individual study results and weights as well as overall weighted mean estimates. Subgroup analysis was performed and to check the heterogeneity meta-regression analysis was done on different headings. Sensitivity analysis was also done to check the robustness of studies.

Publication bias was evaluated by visual inspection of the funnel plot and Egger’s test. We used the Duval and Tweedie trim and fill method to calculate the adjusted effect size accounting for potential publication bias in one of the analyses. A *P*-value of < 0.05 was considered statistically significant.

### Risk of bias

To assess the risk of bias in individual studies for the primary outcome, a standardized critical appraisal instrument, the Cochrane Collaboration’s risk of bias tool (https://training.cochrane.org/handbook/current) was used for RCT. While the Newcastle Ottawa scale (NOS) for the observational study was used for observational studies (http://www.ohri.ca/programs/clinical_epidemiology/oxford.asp). Two reviewers (SK and SS) independently assessed the risk of bias based on sequence generation, allocation concealment, blinding of participants’ personnel and outcome assessors, incomplete outcome data, selective outcome reporting, and other sources of bias. Disagreements were resolved by discussion.

## Results

### Search results and study characteristics

**Fig. 1:**
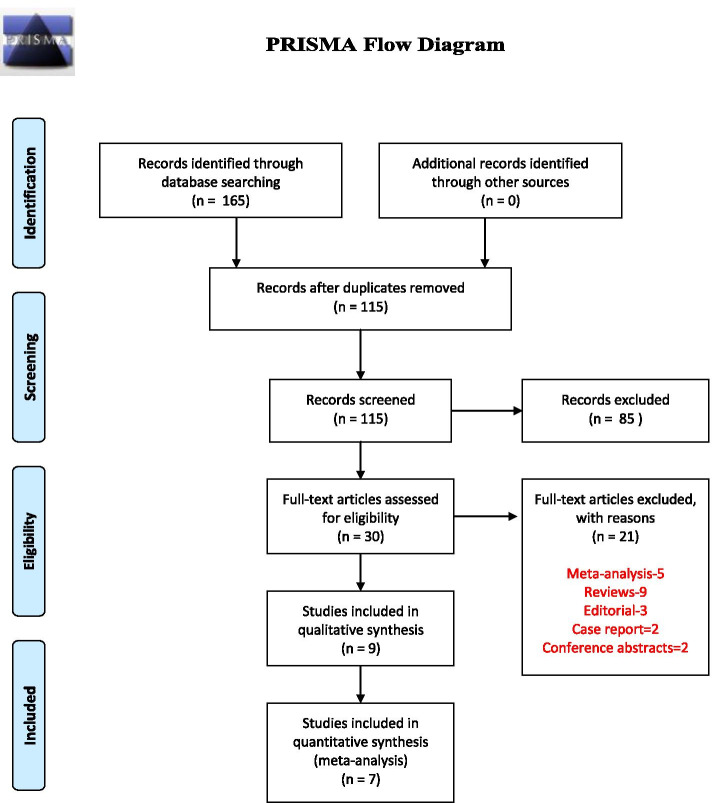
Prisma diagram showing the selection and identification of study

Altogether, 165 studies were obtained from electronic database searches. Out of this, 115 studies were screened by title and abstract after removal of duplicates. The remaining 30 full-text articles were then assessed as per the eligibility criteria. Finally, only 9 studies with a total of 202 patients were included in our study (Fig. 1).

The characteristics of the patients included in our analysis are summarized in Table 1. The included studies consisted of six retrospectives observational studies [13–19], and three randomized controlled trials [19–21]. Of these, only 2 studies used Satralizumab [20, 21], while the remaining seven studies used Tocilizumab [13–18]. The studies were done in different parts of Asia, Europe, and North America. The sample size of the patients ranged from 3 to 63 with female predominance with mean age ranging from 29.4 years to 50 years. The average follow-up duration ranged from 12 months to 31.8 months. The most commonly used dose was 8 mg/kg for 4 weeks intravenously and 120 mg subcutaneously in specified dosage pattern. Add-on drugs were used in all the observational studies, of which the most common were azathioprine and mycophenolate mofetil while in RCTs, only one study assessing monotherapy used no placebo drugs [21].

**Table 1:** Detail characteristics of studies included

Authors	Year	IL-6 inhibitors used	Type of study	Regions/Countries	Sample Size	Follow-up	Sex (Female/Male)	Mean age/Range	Mean disease Duration	AQP-4 ab positive	Dose of drugs used	Add on drugs/Previously used drugs
Ayzenberg	2013	Tocilizumab	Retrospective study	Germany	3	18 months	3/0	39 years (26–40 years)	8.2 years (2.5–9.4 years)	3 (100%)	6 mg/kg every 6 weeks in patients 1 and 2 and every 4 weeks in patient 3	interferon beta-1a and 1b (*n* = 2), glatiramer acetate (*n* = 1), azathioprine (*n* = 1), and mitoxantrone hydrochloride (n = 1). Rituximab
Araki	2014	Tocilizumab	Pilot study	Japan	7	12 months	6/1	12–60 years	NA	7 (100%)	A monthly dose (8 mg/kg)	oral prednisolone, (PSL) and immunosuppressants, including azathioprine
Ringelstein	2015	Tocilizumab	Retrospective Study	Germany	8	30.9 + −15.9 months	8/0	29.4 years (25–49 years)	7.9 + −7.7	8 (100%)	4 of 8 patients at a reduced dosage of 6 mg/kg in 4-week to 6-week intervals, for the other 4 patients dosage of 8 mg/kg at regular 4-week intervals.	immunosuppressant medications, including interferon beta-1b (*n* = 3), interferon beta-1a (n = 2), azathioprine (*n* = 2), mitoxantrone (n = 2), mycophenolate mofetil (n = 1), natalizumab (n = 1), glatiramer acetate (n = 1), alemtuzumab (n = 1), and monthly intravenous corticosteroids
Guarnizo	2018	Tocilizumab	Retrospective Study	Spain	5	NA	3/2	50 + −5.3 years	2.3 years	2 (40%)	8 mg/kg body weight every 4 weeks	Immunosuppressants like rituximab(n = 5), cyclophosphamide(n = 2) and azathioprine (1)
Lotan	2019	Tocilizumab	Retrospective study	USA	12	31.8 + − 18.8 months	11/1	46.9 + − 14.5 (26–68) years	6.8 + − 4.6 years	7 (58.3%)	subcutaneous dose of 162 mg every 1–2 weeks	Corticosteroids, mycophenolate mofetil, intravenous immunoglobulins
Rigal	2020	Tocilizumab	Retrospective study	France	4	23 months	4/0	35.75 years (20–63 years)	7.85 (2–15 years)	4 (100%)	8 mg/kg for monthly intravenous cycles and 162 mg weekly for subcutaneous injections	MMF, Rituximab, Azathioprine
Zhang	2020	Tocilizumab	Randomized Controlled Trial	6 centers in China	59	60 weeks	55/4	48.1 + −13.4 years	6 + −2.9 years	50 (85%)	intravenous tocilizumab 8 mg/kg every 4 weeks.	Azathioprine (AZA) in placebo group
Yamamura	2019	Satralizumab	Randomized Controlled Trial	34 centers in 11 countries	41	NA	37/4	40.8 + −16.1 years (13–73 years)	NA	27 (66%)	dose of 120 mg administered subcutaneously at weeks 0, 2, and 4 and every 4 weeks	AZA, mycophenolate mofetil, glucocorticoids in both groups.
Traboulsee	2020	Satralizumab	Randomized Controlled Trial	44 centers in 13 countries	63	NA	46/17	45.3 ± 12.0 (21–70 years)	NA	41 (65%)	120 mg subcutaneously at weeks 0, 2, 4, and every 4 weeks	None

NOS scale used for observational studies [13–18] found the score ranging from 5 to 7. All the studies were included in the analysis. While for RCTs, two trials [20, 21] had a low risk of bias while the remaining trial [19] had a high risk of bias under the domain deviation from the intended intervention and unclear bias under the domain missing outcome data. (Appendix 2 and 3supplementary file).

### Efficacy outcomes

We carried out our analysis for only one Interleukin-6-receptor inhibitor drug (Tocilizumab) and discuss its efficacy and adverse effects outcome with those in trials of Satralizumab.

### Proportions of relapse-free patients

**Fig. 2:**
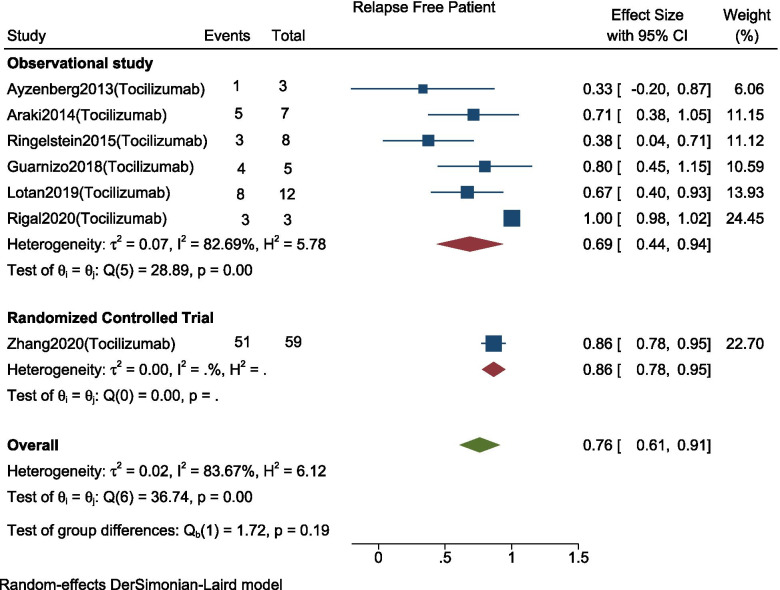
Forest plot with 95% CI for meta-analysis of proportion of patients who were relapse free. The area of each square is proportional to the study’s weight in the meta-analysis, while the diamond shows the pooled result. The horizontal lines through the square illustrate the length of the confidence interval. The width of the diamond serves the same purpose. The overall meta-analyzed measure of effect is imaginary vertical line passing through diamond. (Subgroup Analysis according to study type)

The events of relapse-free patients before and after Interleukin-6-receptor inhibitors therapy (Tocilizumab) was reported in all seven studies (*n* = 97). As the heterogeneity between studies was high (I^2^ = 83.67%, *p* = < 0.001), we conducted a meta-analysis using a random effect model. Our analysis showed that number of relapse-free patients at follow-up with use of Tocilizumab was 76% (95% CI: 0.61–0.91; *p* < 0.001) among which pooled proportion was 69% (95% CI: 0.44–0.94; I^2^ = 82.69%, *p* = < 0.001) in observational studies (*n* = 38) and 86% (95% CI: 0.78–0.95) in RCTs (*n* = 59), with no subgroup difference (*p* = 0.19) (Fig. 2). The differences in effect size according to the study types, duration of follow-up, percentage of Aquaporin-4 Ab positivity, and site of injection are given in subgroup analysis in Table 2.

**Table 2:** Subgroup Analysis in proportion of relapse free patients

Subgroups (no. of studies)			
Study type	Effect size (95% C.I.)	I^2^	Subgroup difference
Observational Study (6)	68.6% (95% CI: 0.44–0.94), p < 0.001	82.69%	0.19
Randomized Controlled Trial (1)	86.4% (95% CI: 0.78–0.95), *p* < 0.001	NA	
Duration of follow-up			
< 20 months (3)	73.9% (95% CI:0.49–0.98), *p* < 0.001	53.54%	0.94
> 20 months (3)	70.8% (95% CI:0.33–1.09), *p* < 0.001	89.55%	
Percentage of AQP-4 positivity			
All (100%) (4)	64.6% (95% CI:0.27–1.02), *p* = 0.001	86.32%	0.31
Not All (100%) (3)	84.3% (95% CI: 0.76–0.92), *p* < 0.001	0%	
Site of Injection			
Intravenous (5)	67.1% (95% CI:0.45–0.89), *p* < 0.001	64.68%	*p* = 0.001
Subcutaneous (1)	100% (95% CI:0.98–1.02), *p* < 0.001	NA	
Mixed (1)	66.7% (95% CI:0.40–0.93), *p* < 0.001	NA	

To explore the possible cause of heterogeneity, meta-regression was done, which showed significant correlation between the outcome and following variables: study types (*p* < 0.001), duration of follow up (*p* = 0.003) and site of injection (*p* < 0.001). While a non-significant correlation was found between the outcome and the percentage of AQP-4 positivity (*p* = 0.208). The information for meta-regression analysis is given in Table 3.

**Table 3:** Meta Regression for subgroups of relapse free patients

Variables	Coef.	Std. Err.	z	P > |z|	[95% Conf. Interval]
Study type	−0.7796787	0.1653799	−4.17	< 0.001	−1.103817 .920804
Duration of followup	−0.208344	0.0071029	−2.93	0.003	−.0347557–.006913
AQP-4 positivity	0.0030413	0.0024175	1.26	0.208	−.0016968 .0077795
Site of injection	0.5235	0.1142147	4.58	< 0.001	.2996432 .7473567
_cons	0.6510876	0.2339978	2.78	0.005	−.1924603 1.109715

Sensitivity analysis done showed stable overall effect size after testing for all study omitted. The inspection of the funnel plot and egger’s test (*p* = < 0.001) showed significant publication bias. (Supplementary Fig. 1). The adjusted proportion using the Duval and Tweedie trim and fill method was 91.9% of patients (95% CI: 0.79–1.05, 4 studies imputed).

### Change in ARR

Changes in ARR before and after Interleukin-6-receptor inhibitor therapy (Tocilizumab) was reported in 5 studies (*n* = 35). For those studies who did not report mean and standard deviation (SD), individual data, mean and SDs were calculated, and in studies reporting as a median, range, and interquartile range, it was converted into mean and SD [22].

**Fig. 3:**
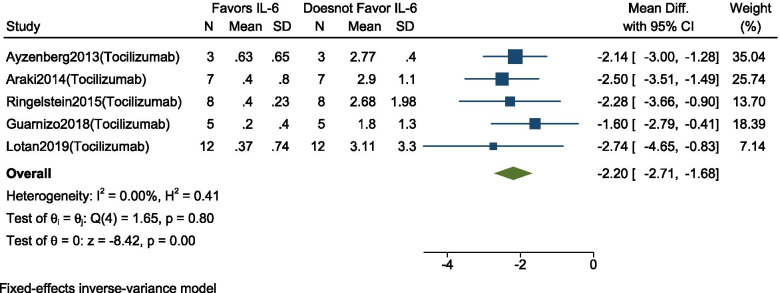
Forest plot with 95% CI for meta-analysis of efficacy on the mean ARR reduction. The square shows the mean difference for each study. The diamond at the bottom of the graph shows the average effect size of included studies

As there was no heterogeneity between the studies (I^2^ = 0%, *p* = 0.80), we conducted a meta-analysis using a fixed-effect model. Our analysis showed that the use of this therapy significantly reduced ARR at follow-up by 2.6 (95% CI: − 2.71 to − 1.68; *p* < 0.001) (Fig. 3). Publication bias was not conducted because of a small number of studies.

### Change in EDSS score

**Fig. 4:**
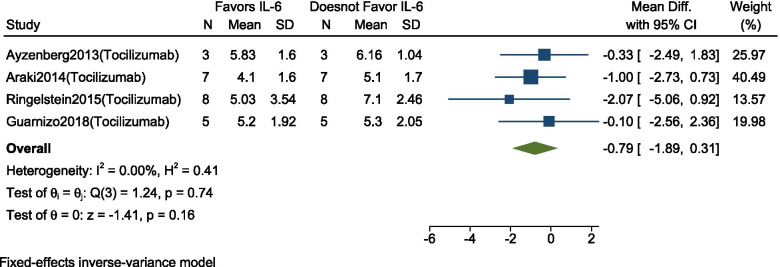
Forest plot with 95% CI for meta-analysis of efficacy on the mean EDSS reduction. The square shows the mean difference for each study. The diamond at the bottom of the graph shows the average effect size of included studies

Changes in EDSS before and after Interleukin-6-receptor inhibitor therapy (Tocilizumab) was reported in 4 studies (*n* = 23). As there was no heterogeneity between the studies (I^2^ = 0%, *p* = 0.74), we conducted a meta-analysis using a fixed-effect model. Our analysis showed that this therapy group did not significantly influence EDSS scores at follow-up. (MD = -0.79, 95% CI: − 1.89 to − 0.31; *p* = 0.16) (Fig. 4). Considering a small number of studies, Publication bias was not conducted.

### Safety outcomes

#### Proportions of patients with adverse events

**Fig. 5:**
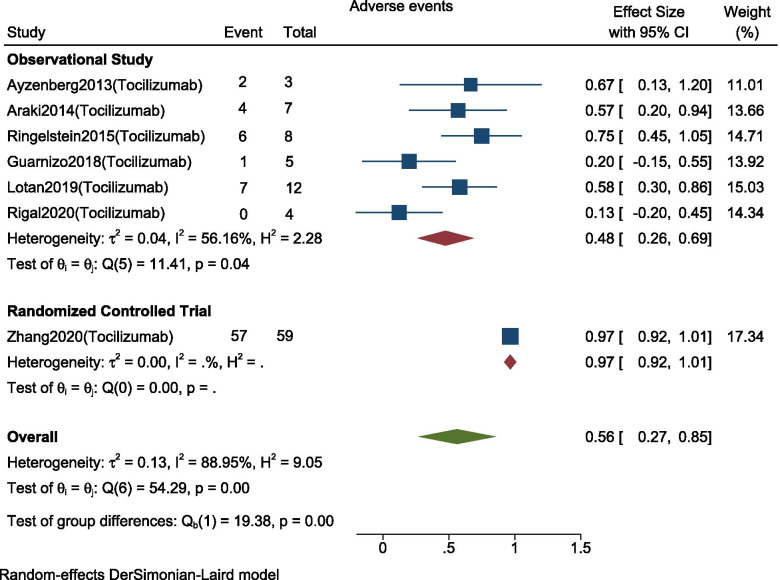
Forest plot with 95% CI for meta-analysis of proportion of patients who experienced adverse effects. The area of each square is proportional to the study’s weight in the meta-analysis, while the diamond shows the pooled result. The horizontal lines through the square illustrate the length of the confidence interval. The width of the diamond serves the same purpose. The overall meta-analyzed measure of effect is imaginary vertical line passing through diamond

The pooled proportions of patients with adverse events in studies using Tocilizumab (*n* = 98) were 56% (95% C.I.;0.27–0.85, I^2^ = 88.95%, *p* < 0.001) among which 48% (95% C.I.;0.26–0.69, I2 = 56.16%, *p* = 0.04) was in observational studies and 97% (95% C.I.;0.92–1.01) in RCTs with significant subgroup difference (*p* = < 0.001). (Fig. 5).

Adverse events were reported among (*n* = 198) patients in all nine studies. Most common adverse effects were upper respiratory tract infections (*n* = 49), urinary tract infections (*n* = 43), hypercholesterolemia (*n* = 13), leucopenia (*n* = 12), fatigue (*n* = 20), and anemia (*n* = 19).

### Proportions of patients with serious adverse events

**Fig. 6:**
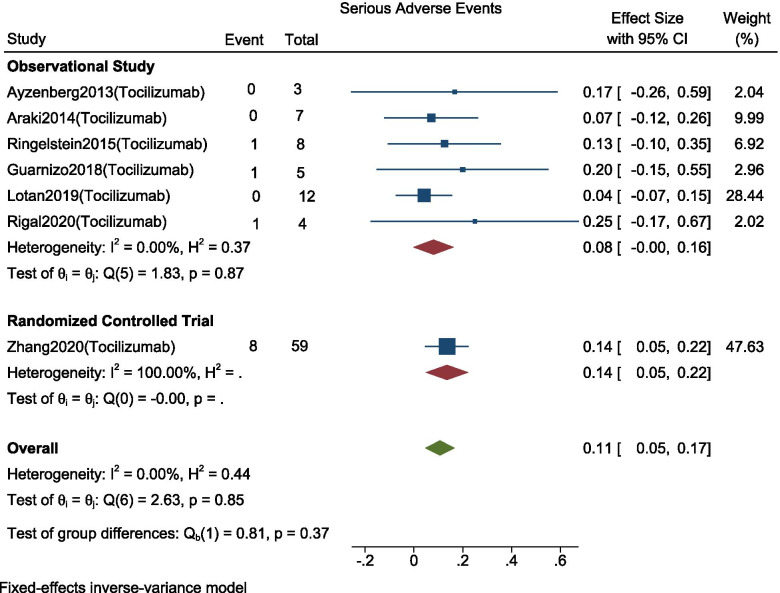
Forest plot with 95% CI for meta-analysis of proportion of patients who experienced serious adverse effects. The area of each square is proportional to the study’s weight in the meta-analysis, while the diamond shows the pooled result. The horizontal lines through the square illustrate the length of the confidence interval. The width of the diamond serves the same purpose. The overall meta-analyzed measure of effect is imaginary vertical line passing through diamond

Serious adverse events are those that interrupt the patient’s daily activities and may lead to systemic medication or other treatment. The pooled proportions of patients with serious adverse events for studies using Tocilizumab (*n* = 98) were 11% (95% C.I.; 0.05 to 0.17, I^2^ = 0%, *p* < 0.001) and a significant subgroup difference was not seen based on study type (*p* = 0.37). (Fig. 6).

Serious Adverse events were reported among (*n* = 180) patients in six studies among all nine studies. Bacterial infections like pneumonia and Deep Vein Thrombosis were the common serious adverse events.

### Mortality

Two patients died in two studies, both in the Tocilizumab group. One with cervical myelitis and another with relapse of longitudinally extensive transverse myelitis. Both the deaths were not related to the treatment complications.

## Discussion

To the best of our knowledge, this is the first meta-analysis to evaluate the effectiveness and safety of Interleukin-6-receptor inhibitors in the treatment of NMOSD.

Satralizumab and Tocilizumab are humanized monoclonal antibodies targeting IL-6 receptor or IL-6 which act by promoting differentiation of inflammatory cells inducing morbific antibodies production in NMOSD as a pro-inflammatory cytokine [20]. With the use of antibody recycling technology, Satralizumab has better pharmacokinetics than Tocilizumab [23]. CSF and serum IL-6 levels are found to be increased in patients with NMOSD. IL-6 promotes plasmablast survival, stimulating the secretion of AQP-4 IgG, reducing blood-brain barrier (BBB) integrity and functionality, and enhancing proinflammatory T-lymphocyte differentiation and activation; a driving factor for disease severity in NMOSD. Thus, IL-6 inhibition is now being considered to improve disease severity and control [24]. Among them, tocilizumab is found to have a shorter dosing interval than Satralizumab. Similarly, Satralizumab was tested both as a monotherapy versus placebo and in combination with basic therapeutic agents [25].

Barros et al. showed a correlation between baseline serum IL-6 levels and risk of relapses and severity. During a 2-year disease follow-up period in these patients, an increase of 8-fold relapse risk was observed in patients with IL-6 serum concentrations above baseline during remissions [26]. Uzawa et al. found patients with high CSF IL-6 levels to have shorter relapse-free duration than with low levels after relapse (*p* = 0.079) [27]. Similarly, it also found an only modest improvement in disability of patients with high IL-6 levels [27].

Our analysis showed a promising result. We found that following Interleukin-6-receptor inhibitor (Tocilizumab) therapy, a significant proportion of patients was relapse-free (76%), mean ARR reduced by 2.6 at follow-up but not a significant decrease in EDSS at follow-up among treatment groups.

SAkura Sky (Satralizumab in combination with baseline immunosuppressants) and SAkura Star (Satralizumab monotherapy) are two trials assessing efficacy and safety of Satralizumab [20, 21].

In SAkura Star trial, 30% of patients receiving Satralizumab had a protocol-defined relapse as compared to 50% of the patient’s receiving placebo, and in SAkura Sky trial, 20% receiving Satralizumab had a protocol-defined relapse, as compared to 43% of patients receiving placebo. Thus, the relapse-free period in the above two studies among patients receiving Satralizumab was 70% and 80% respectively. These figures are comparable to the tocilizumab group in this analysis.

In SAkura Sky, The ARR during the double-blind period was 0.11 in the Satralizumab group and 0.32 in the placebo group. While the change in EDSS score (*n* = 29) at 24 weeks was − 0.10. In SAkura Star, the change from baseline for ARR was 0.17 (0.10 to 0.26) and EDSS was − 0.34 (− 0.62 to − 0.05). In both cases, ARR and EDSS decreased from baseline on use of IL-6 receptor inhibitors as compared to placebo [20, 21]. In comparison to our results for Tocilizumab, ARR was significantly reduced while reduction of EDSS at follow-up was insignificant.

A recent meta-analysis of Xue et al. analyzed the safety and efficacy of different monoclonal antibodies used in NMOSD among RCTs. Sub-group analysis of this study found a significant decrease in on-trail relapse risk and EDSS at follow-up (analysis of two trials of Satralizumab) but non-significant difference in mean ARR among the treatment groups and placebo group. IL-6 inhibitors were also found to be superior to other monoclonal antibodies in reducing EDSS [28]. A meta-analysis describing the safety and efficacy of Tocilizumab has similar therapeutic outcomes as compared to our analysis considering results in a change in mean ARR and EDSS score following treatment as only more studies of Tocilizumab treatment were added in our analysis [29].

Between the subgroups of study type (Observational studies and RCTs), our analysis found no significant subgroup difference in efficacy outcomes. Individual trail [20, 21] has shown significant reduction in relapses for AQP-4 Ab positive patients in comparison to AQP-4 Ab negative patients and significant increase with duration of treatment/follow up but in our analysis mean duration of follow up and percentage of AQP-4 positivity neither had a significant subgroup difference on the effectiveness of therapy. However, considering the route of administration of the drug (intravenous vs subcutaneous), studies with subcutaneous injection found differing proportion of relapse-free patients than those with intravenous administration. This finding cannot be generalized because both have been used inconsistently. But, a study by lotan et al. found subcutaneous injections equally effective as IV formulations while subcutaneous injections more advantageous due to ease of in-home administration [17].

With the use of Tocilizumab, the pain levels decreased after six and 12 months of treatment in seven patients in the study by Araki et al., more at 12 months of treatment [14]. While, Ringelstein et al. for Tocilizumab showed decreased pain levels from a median of 6.5 to 2.5, at last, follow up among the eight patients [15]. Araki et al. also showed decreased general fatigue at 6 and 12 months of follow-up as compared to treatment initiation [14]. For Satralizumab, SAkura Sky trial showed an insignificant between-group difference in the change in the mean visual analog scale (VAS) pain score and mean functional assessment of chronic illness therapy-fatigue (FACIT-F) score [20]. Also, the change of VAS pain score and FACIT-F score from baseline in the SAkura Star, was non-significant [21]. The probable reason could be that Satralizumab had little effect on the average VAS pain score. Additional factors like the heterogeneity of pain syndromes, use of concomitant medications for pain might play some role.

In terms of safety issues, the proportion of patients with adverse events and serious adverse events for Tocilizumab was 56 and 11% respectively. While in the trials for Satralizumab, adverse events and serious adverse events were 37(90%) and 7(17%) for SAkura Sky while 58(92%) and 12(19%) for SAkura Star respectively [20, 21]. Though there is difference in the frequency but, most of the common side effects are similar. The frequency of most common adverse events like Upper respiratory tract infections, Urinary Tract Infections, hypercholesterolemia, and serious adverse events are similar to studies by Xie et al. and Xue et al. [28, 29] Though, cardiovascular disease is the main safety of concern in Anti-Interleukin-6 receptor inhibitors like Satralizumab and Tocilizumab as a result of an increase in cholesterol levels; recent trials [20] have shown no increase in the risk of cardiovascular disease [30]. No mortality was observed in two trials of Satralizumab in comparison to two treatment related deaths in Tocilizumab used studies. Most of the adverse events in these studies were caused by drug effect and accidental occurrence mainly during relapse and there was a very small mortality rate. These evidences suggest that Interleukin-6-receptor inhibitors therapy is safe and well-tolerated with an acceptable adverse effects profile.

Recently, Satralizumab has been approved by the US Food and Drug Administration (FDA) for the treatment of NMOSD based on two RCTs; SAkuraSky and SAkuraStar trial. Canada also approved subcutaneous Satralizumab for the treatment of NMOSD in adults and children aged ≥ 12 years with AQP-4 seropositivity [31]. While Tocilizumab is still used off-label in some case studies and in clinical studies. Tocilizumab, however, is considered a safe and effective alternative to azathioprine in controlling relapses with the need for further trials [19]. Comparison of safety and efficacy of Satralizumab and Tocilizumab was not effective in our study, as ideally, head-to-head trials should be conducted for direct comparative analysis and evaluation. Though, Interleukin-6-receptor inhibitors have established themselves as an important class of monoclonal antibodies in the field of treatment of relapses of NMOSD, the road ahead is long, as the benefits are only applicable to a large subset of AQP4-Ab seropositive patients leaving behind the important hurdle to find a drug that can impact the disease course of AQP-4 Ab seronegative groups [10].

Our meta-analysis has several strengths. We have systematically collected all evidence including real-world data and RCTs for the efficacy and safety of Interleukin-6-receptor inhibitors. Though, our study included 9 studies with 202 patients receiving Interleukin-6-receptor inhibitors but analysis was only done for seven studies which used Tocilizumab. Errors in the calculation of data used in the previous meta-analysis were rectified, if present. The main limitation of our analysis is heterogeneity among studies in two analyses; the proportion of Relapse free patients and adverse events with publication bias in the initial one. Variability in sample size, follow time, drug use, AQP-4 positivity rate, and sites of injection causing heterogeneity is another limitation. The use of different add-on drugs like immunosuppression to reduce relapses may also add on to heterogeneity. In addition, like previous studies, though effective and with acceptable adverse effects, the role of tocilizumab as a first-line disease-modifying therapy still remains to be explored.

## Conclusions

Our meta-analysis showed Tocilizumab has significant benefits in reducing mean ARR and increasing the number of relapse-free patients with acceptable adverse events profiles. The similar efficacy outcomes and favorable safety profiles were found for Satralizumab in two trials. However, data on chronic pain and fatigue were contrasting. Thus, more long-term trials and placebo-controlled trials including large subsets of both AQP4-Ab seropositive and AQP4-Ab seronegative NMOSD patients are needed.

## Supplementary Information

**Additional file 1: Appendix 1:** Search strategy used in the current systematic review and meta-analysis. **Appendix 2:** Newcastle-Ottawa Scale for assessing quality of non-randomized/observational studies. **Appendix 3:** Cochrane Collaboration tool for assessing quality of randomized controlled trials.

**Additional file 2: Supplementary Figure 1** (A): Funnel plot for detection of publication bias in meta-analysis of proportion of patients with relapse free events. Black dots represent imputed studies and brown dot represent added studies for trim and fill. (Without trim and fill B). (B): Funnel plot for detection of publication bias in meta-analysis of proportion of patients with relapse free events. Black dots represent imputed studies and brown dot represent added studies for trim and fill. (With trim and fill).

## Data Availability

All the necessary data and information are within the article. Supplementary file with the search strategy has been provided.

## Abbreviations

IL-6: Interleukin-6
NMOSD: Neuromyelitis Optica spectrum disorder
ARR: Annualized Relapse ratio
EDSS: Extended Disability Status Scale
MD: Mean difference
RCT: Randomized Controlled Trial
AQP4-IgG: Aquaporin-4-Immunoglobulin G
MOG: Myelin oligodendrocyte glycoprotein
NOS: Newcastle Ottawa scale
SD: Standard Deviation
BBB: Blood Brain Barrier
FDA: Food and Drug Administration
VAS: Visual Analog Scale
FACIT-F: Functional Assessment of Chronic Illness Therapy-Fatigue
