# Supplementary material for: Safety and efficacy of interleukin-6-receptor inhibitors in the treatment of neuromyelitis optica spectrum disorders: a meta-analysis
Source: BMC Neurol. 2021 Nov 23;21:458. doi: 10.1186/s12883-021-02488-y (PMC8609802; doi:10.1186/s12883-021-02488-y)

**Supplementary Content**

**Safety and Efficacy of IL-6 inhibitors in the treatment of NMOSD: A meta-analysis**

**Appendix 1:** Search strategy used in the current systematic review and meta-analysis.

**Appendix 2:** Newcastle-Ottawa Scale for assessing quality of non-randomized/observational studies.

**Appendix 3:** Cochrane Collaboration tool for assessing quality of randomized controlled trials.

This supplementary material has been provided by the authors to give readers additional information.

**Appendix 1:** Search strategy used in the current systematic review and meta-analysis.

**PubMed search strategy:**

#1 "Neuromyelitis Optica "[Mesh] OR "neuromyelitis optica spectrum disorder" OR “NMOSD”

#2 "Anti-interleukin-6” OR ““anti-IL-6” OR”IL6 receptor blockade” OR ”Tocilizumab” OR “Satralizumab”

#3 “randomized controlled trial” [PT] OR “randomized” OR “placebo” OR "Observational study" OR "case series"

#4: #1 AND #2 AND #3

**EMBASE search strategy:**

#1 'Neuromyelitis optica'/exp OR 'Neuromyelitis optica spectrum disorder’

#2 ‘Anti-interleukin-6’ OR ‘anti-IL-6’ OR ‘IL6 receptor blockade’ OR ‘Tocilizumab’ OR ‘Satralizumab’

#3 'placebo'/exp OR placebo OR 'randomized controlled trial':jt OR 'randomized':au OR 'observational' OR "case series'

#4 : #1 AND #2 AND #3

**Cochrane search strategy:**

#1 MeSH descriptor: [Neuromyelitis Optica] explode all trees

#2 Neuromyelitis optica spectrum disorder

#3: #1 OR #2

#4: (Anti-interleukin-6) OR (anti-IL-6) OR (IL6 receptor blockade) OR (Tocilizumab) OR (Satralizumab)

#5: #3 OR #4

#6 : #2 AND #5

**Appendix 2:** Newcastle-Ottawa Scale for observational studies (<http://www.ohri.ca/programs/clinical_epidemiology/oxford.asp>)

| Study Name | Selection | Comparability | Outcome | Total Score | Risk of Bias | Included/ Excluded |
| --- | --- | --- | --- | --- | --- | --- |
| Ayzenberg 2013 et al. | 2 | 1 | 2 | 5 | High | Included |
| Araki2014 et al. | 2 | 1 | 3 | 6 | High | Included |
| Ringelstein2015 et al. | 2 | 1 | 3 | 6 | High | Included |
| Gaurnizo2018 et al. | 2 | 1 | 3 | 6 | High | Included |
| Lotan2019 et al. | 2 | 2 | 3 | 7 | Low | Included |
| Rigal2020 et al. | 2 | 1 | 3 | 6 | High | Included |

Note: Mean scores greater or equal to 5 are included in analysis.

**Appendix 3:** Cochrane Collaboration tool for assessing quality of randomized controlled trials.

All studies were assessed for random sequence generation, allocation concealment, blinding of participants, incomplete data outcome, and selective outcome reporting. “-” indicate high risk of bias, “+” indicate low risk of bias and “!” indicate unclear risk of bias or some concerns.


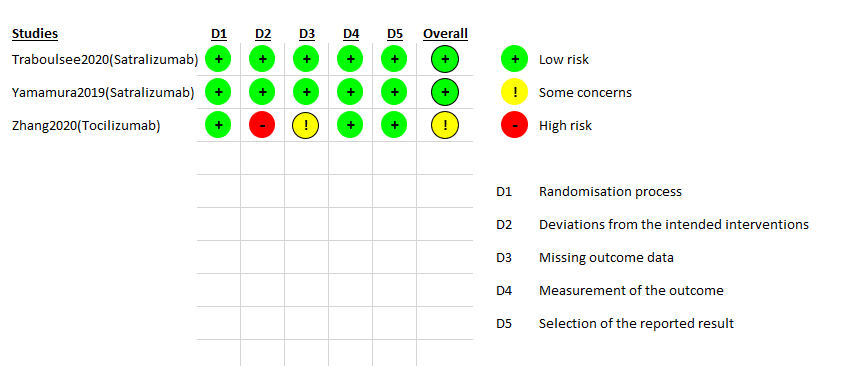

Supplement: Supplementary file 1 — Additional file 1: Appendix 1: Search strategy used in the current systematic review and meta-analysis. Appendix 2: Newcastle-Ottawa Scale for assessing quality of non-randomized/observational studies. Appendix 3: Cochrane Collaboration tool for assessing quality of randomized controlled trials. [file 12883_2021_2488_MOESM1_ESM.docx]
