# Supplementary material for: Safety and efficacy of interleukin-6-receptor inhibitors in the treatment of neuromyelitis optica spectrum disorders: a meta-analysis
Source: BMC Neurol. 2021 Nov 23;21:458. doi: 10.1186/s12883-021-02488-y (PMC8609802; doi:10.1186/s12883-021-02488-y)
Supplement: Supplementary file 2 — Additional file 2: Supplementary Figure 1 (A): Funnel plot for detection of publication bias in meta-analysis of proportion of patients with relapse free events. Black dots represent imputed studies and brown dot represent added studies for trim and fill. (Without trim and fill B). (B): Funnel plot for detection of publication bias in meta-analysis of proportion of patients with relapse free events. Black dots represent imputed studies and brown dot represent added studies for trim and fill. (With trim and fill). [file 12883_2021_2488_MOESM2_ESM.docx]

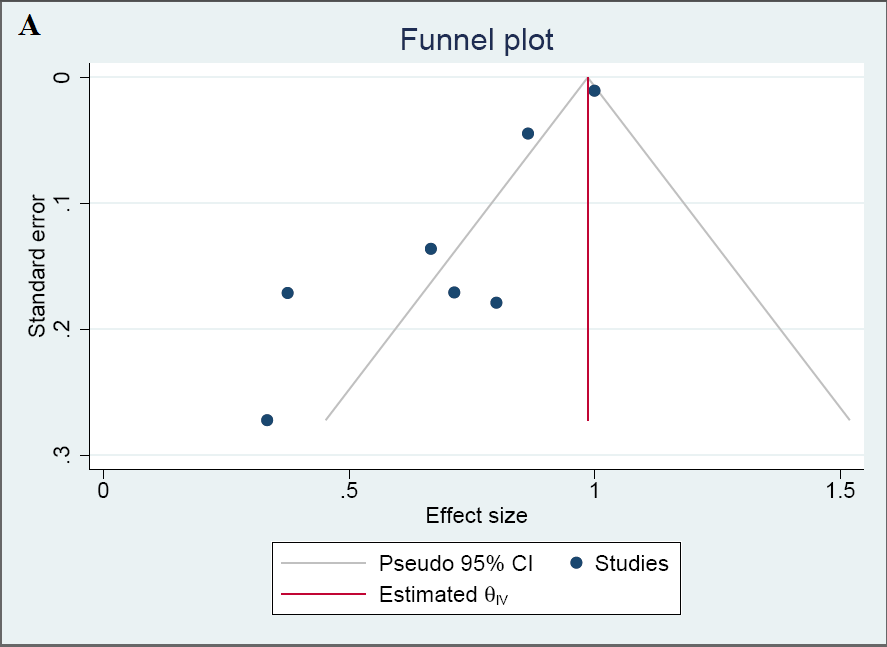


Supplementary Figure 1(A): Funnel plot for detection of publication bias in meta-analysis of proportion of patients with relapse free events. Black dots represent

imputed studies and brown dot represent added studies for trim and fill. (Without trim and fill B)


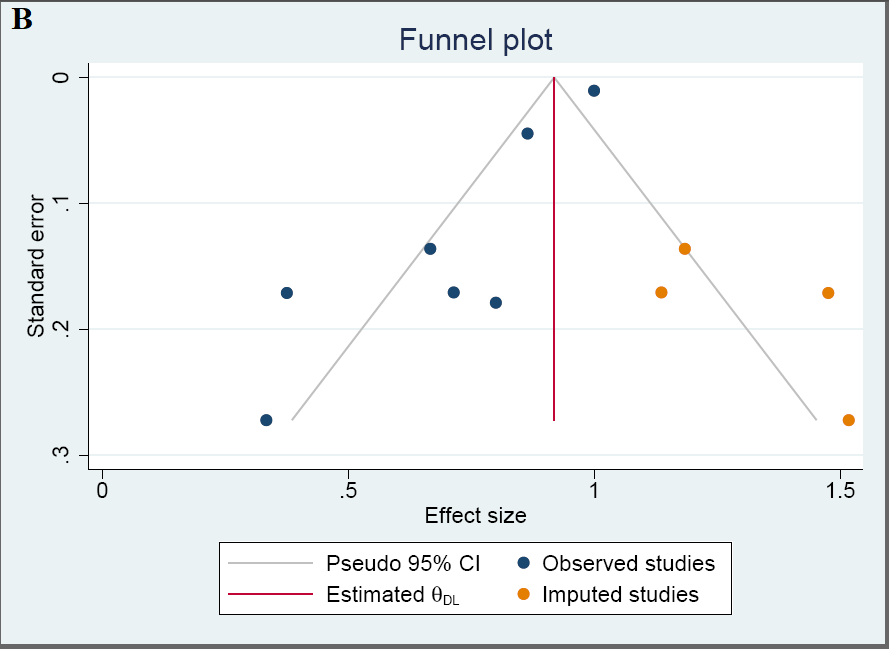


Supplementary Figure 1(B): Funnel plot for detection of publication bias in meta-analysis of proportion of patients with relapse free events. Black dots represent

imputed studies and brown dot represent added studies for trim and fill. (With trim and fill)
